# Supplementary material for: Toward Adoption of Health Risk Assessment in Population-Based and Clinical Scenarios: Lessons From JADECARE
Source: Int J Integr Care. 2024 Jun 4;24(2):23. doi: 10.5334/ijic.7701 (PMC11160407; doi:10.5334/ijic.7701)
Supplement: Supplementary Material. — Toward adoption of health risk assessment in population-based and clinical scenarios. [file ijic-24-2-7701-s1.pdf]

Toward adoption of health risk assessment in population-based and clinical scenarios

Rubèn González-Colom et al.

(On-line supplementary material)

TABLE OF CONTENTS

1 APPENDIX 1 RESULTS OF THE IMPLEMENTATION ... 2

1.1 MARCHE REGION (I) ... 2

1.1.1 Local Action Plan ... 2

1.1.2 Plan-Do-Study-Act Cycles ... 5

1.2 ESTONIA (EE) ... 19

1.2.1 Local Action Plan ... 19

1.2.2 Plan-Do-Study-Act Cycles ... 23

APPENDIX 2: JADECARE THEMATIC WORKSHOP, LECTURE ON HEALTH RISK ASSESSMENT, DAVID MONTERDE - VILJANDI 14TH JUNE 2022 ... 39

1 APPENDIX 1 RESULTS OF THE IMPLEMENTATION

1.1 MARCHE REGION (I)

1.1.1 Local Action Plan

1.1.1.1 Local Good Practice

<table>
<tr><th>Local Good Practice</th><td colspan="2">A stratification tool for an effective management of chronic diseases in the Marche region</td></tr>
<tr><th colspan="2">Target population</th><th>Setting(s)</th></tr>
<tr><td colspan="2">The entire population of Marche region (~1,500,000 habitants)</td><td>The Regional Health System</td></tr>
<tr><th colspan="3">Main aim</th></tr>
<tr><td colspan="3">To apply a population stratification tool to improve the efficiency of the Regional Health System and the quality of life of citizens by providing services that meet their needs.</td></tr>
<tr><th>Outcomes</th><th>Local Core Features and their Components</th><th>Inputs</th></tr>
<tr><td>• Providing an in-depth analysis of the health status of the Marche population
• Guaranteeing continuity of care throughout the region
• Reorganising services based on the health needs of people suffering from chronic diseases.
• Assessing the economic impact of the reorganisation of services for chronic diseases.</td><td>Implement a risk stratification tool based on adjusted morbidity groups (LCF1)
• criteria for local stratification model
• data extraction and processing mechanisms
• definition of care programs for each of the strata
Build a map/dashboard of citizens’ health/risk and available services (LCF2)
• City/village/district indicators based on the GMA and/or chronic diseases.
• Map available services in each area
• dashboard for data visualization</td><td>• Data
• Staff
• IT system
• Funding
• Decision-makers
• Technical assistance</td></tr>
<tr><th colspan="3">General description</th></tr>
<tr><td colspan="3">The intervention consists of setting up and testing a stratification tool for planning and decision-making purposes.</td></tr>
<tr><th colspan="3">Local Core Feature 1</th></tr>
<tr><td colspan="3">Component 1 is represented by the Catalan GMA population stratification algorithm, suitably adapted to the regional context and available health data. This tool will focus on chronic diseases and will make it possible to assess the population of the Marche Region placed in the higher sections of the Kaiser pyramid.</td></tr>
<tr><th colspan="3">Local Core Feature 2</th></tr>
<tr><td colspan="3">Component 2 envisages displaying on Marche region map the aggregated data from the stratification (and other indicators related to chronic diseases) as well as the available services to facilitate analysis and planning activities.</td></tr>
</table>

1.1.1.2 Local Action Plan

<table>
<tr><td colspan="2">Local Good Practice</td><td colspan="4">A stratification tool for an effective management of chronic diseases in the Marche region</td></tr>
<tr><td colspan="4">Target population</td><td colspan="2">Setting</td></tr>
<tr><td colspan="4">The entire population of Marche region (~1,500,000 habitants)</td><td colspan="2">The Regional Health System</td></tr>
<tr><td colspan="6">Main aim</td></tr>
<tr><td colspan="6">To apply a population stratification tool to improve the efficiency of the Regional Health System and the quality of life of citizens by providing services that meet their needs.</td></tr>
<tr><td colspan="6">General description</td></tr>
<tr><td colspan="6">The intervention consists of setting up and testing a stratification tool for planning and decision-making purposes.</td></tr>
<tr><td>Related oGPs and CFs</td><td colspan="5">Catalan Open Innovation Hub on ICT
Block 1: Health Risk Assessment:
CF1.1 Assessment of transferability, and identification of steps for adoption, according to intellectual property rules, of the Catalan population-based risk stratification tool (AMG) into the ecosystem of the next adopter.
CF1.2 Health data management strategies
CF1.3 Development of enhanced risk prediction modelling for health policy purposes and/or clinical risk prediction.</td></tr>
<tr><td colspan="2">Local Core Feature 1</td><td colspan="4">Implement a risk stratification tool based on adjusted morbidity groups (AMG)</td></tr>
<tr><td colspan="6">SMART objective</td></tr>
<tr><td colspan="6">In the framework of JADECARE, Marche Region will set up a risk stratification tool based on GMA, which will support healthcare services programming, with a focus on chronic diseases.</td></tr>
<tr><td>Activities</td><td>Actors</td><td>Resources</td><td>Setting(s)</td><td>Timeline</td><td>KPIs</td></tr>
<tr><td>Identify criteria for local stratification model</td><td>-Healthcare professionals
-IT experts
-Data scientists</td><td>Catalan GMA</td><td>Marche Region/Regional Health Agency</td><td>3 months (December 2021-February 2022)</td><td>List of criteria (Y/N)</td></tr>
<tr><td>Implement data extraction and processing mechanisms</td><td>-Healthcare professionals
-IT experts
-Data scientists</td><td>-Health data
-IT infrastructure
-Software</td><td>Marche Region/Regional Health Agency</td><td>6 months (March-August 2022)</td><td>-Database with required health data (Y/N)
-Availability of IT infrastructure (Y/N)
-% of data processed</td></tr>
<tr><td>Define care programs and services for each of the strata</td><td>-Healthcare professionals
-Marche/ARS staff</td><td>NA</td><td>Marche Region/Regional Health Agency</td><td>4 months (July-September 2022)</td><td>Short report on care programs and services</td></tr>
<tr><td colspan="2">Local Core Feature 2</td><td colspan="4">Build a map/dashboard of citizens' health/risk and available services</td></tr>
<tr><td colspan="6">SMART objective</td></tr>
<tr><td colspan="6">In the framework of JADECARE, Marche Region will develop a map/dashboard allowing in-depth analysis of stratification data and available services for policy- and decision-making processes</td></tr>
<tr><td>Activities</td><td>Actors</td><td>Resources</td><td>Setting(s)</td><td>Timeline</td><td>KPIs</td></tr>
</table>

Definition of indicators on stratification and/or chronic diseases	-Healthcare professionals<br>-IT experts<br>-Data scientists<br>-Marche/ARS staff	NA	Marche Region/Regional Health Agency	2 months (September-October 2022)	List of indicators (Y/N)
Mapping of available services for chronic diseases in Marche Region	-Healthcare professionals<br>-Marche/ARS staff	Data on available services	Marche Region/Regional Health Agency	2 months (September-October 2022)	List of services (Y/N)
Set up of the dashboard for data visualization	-Healthcare professionals<br>-IT experts<br>-Data scientists<br>-Marche/ARS staff	Subcontract for the dashboard	Marche Region/Regional Health Agency	6 months (June-November 2022)	Availability of the dashboard (Y/N)
Identification of policies and interventions at regional level to support implementation and sustainability of the LGP	-Project manager<br>-Marche/ARS staff<br>-Regional policy representatives	NA	Marche Region/Regional Health Agency	2022-2023	List of policies and interventions (Y/N)

1.1.2 Plan-Do-Study-Act Cycles

1.1.2.1 1st PDSA Cycle

1.1.2.1.1 Plan

LCF1	Implement a risk stratification tool based on adjusted morbidity groups (AMG)							
**Activities**	**Actions**	**Actors**	**Timeline**	**KPIs measure (data collection)**				
				**KPI**	**Who**	**When**	**How**	**Target**
Activity 1: Identify criteria for local stratification model	Define the **cohort study** based on chronicity, comorbidities and vulnerability<br><br>Define sources of stratification criteria:<br><br>1.ICD-9CM and ICD-10CM, from administrative health databases: Drugs, Hospital admission, Emergency and Urgency, Homecare, Exemption, Mortality registry.<br><br>2. National and regional criteria of extremely frail and vulnerable patients, in Covid-vaccination booking<br><br>Analysis of algorithm's preliminary results (strings' control and data validation)	-Computer specialist<br><br>-IT experts<br><br>-Healthcare professional<br><br>-Data scientists	1/12/21 to 31/03/22	List of criteria used for the stratification (Y/N)	Project Manager	31/03/22	Utilising information technology systems<br><br>Monitoring during the monthly follow-up meetings	Yes
Activity 2:	Standardize variables format based on	-Computer	01/03/22	-Database with	Project	30/04/22	Utilising	Yes

Implement data extraction and processing mechanisms	algorithm for stratification	specialist<br>-IT experts<br>-Healthcare professional<br>-Data scientists	to 30/04/22	required health data (Y/N)<br>-Availability of IT infrastructure (Y/N)<br>-% of data processed	manager		information technology systems<br>Monitoring during the monthly follow-up meetings	
	Automating the data extraction and validation process		01/05/22 to 31/07/22			31/07/22		
	Implement algorithm tool		01/08/22 to 31/08/22			31/08/22		
Activity 3: Define care programs and services for each of the strata	Elaborate population management strategies for GMA 1,2,3,4, starting with health promotion, to case management strategy, focusing on chronic diseases management	-Healthcare professional<br>-Marche/ARS staff	31/07/22 to 30/09/22	Short report on care programs and services (Y/N)	Project manager	30/09/22	Relying on specific evaluation and guide from healthcare professionals<br>Monitoring during the monthly follow-up meetings	Yes

1.1.2.1.2 Do

Cycle number (1 or 2)	1	
**Activity**	**KPI**	**Actual value**
**LCF 1 – Activity 1:** Identify criteria for local stratification model	List of criteria used for the stratification (Y/N)	Yes
**LCF 1 – Activity 2:** Implement data extraction and processing mechanisms	Database with required health data (Y/N)	No
	Availability of IT infrastructure (Y/N)	N.A.<br>(Expected during PDSA 2)
	% of data processed	N.A.<br>(Expected during PDSA 2)
**LCF 1 – Activity 3:** Define care programs and services for each of the strata	Short report on care programs and services (Y/N)	N.A.<br>(Expected during PDSA 2)

QUESTIONS	ANSWERS		
**What was actually implemented? Any deviation from the planned actions**	Main achievements of PDSA1 are:<br><br>- identification of data sources as input for the GMA<br>- evaluation of the quality of the health data of Marche region<br>- evaluation of the applicability of the GMA in Marche Region<br>- preliminary analysis of the health status of Marche citizens.<br><br>Some deviations occurred: there were some delays due to the internal reorganisation and administrative procedures for personnel recruitment. Transfer of the algorithm and related documentation took more time than expected due to authorization procedures at the oGP site. The order of actions under Activity 2 changed after technical evaluations.		
**Problems? Unexpected findings? Please describe**	Some problems/challenges occurred due to the internal reorganisation and the overburdened personnel. Access and management of the administrative health databases is challenging due to privacy restrictions, authorizations, IT issues. However, these issues are constantly monitored and partially solved.<br><br>Main barrier to the full implementation of the tool is the GDPR application in Italy.<br><br>As regards unexpected findings, we can acknowledge the great results obtained during the pilot test with the GMA.		
**IMPLEMENTATION PROGRESS OF THE LOCAL GOOD PRACTICE**			
**0-25%**	**25-50%**	**50-75%**	**75-100%**
	**x**		

1.1.2.1.3 Study

Cycle number (1or 2)		1				
**Activity**	**KPI**	**Target value**	**Actual value**	**Reasons for the deviations**	**Mitigation actions implemented**	**Impact of mitigation actions**
**LCF 1 – Activity 1:** Identify criteria for local stratification model	List of criteria used for the stratification (Y/N)	Yes	Yes			
**LCF 1 – Activity 2:** Implement data extraction and processing mechanisms	Database with required health data (Y/N)	Yes	No	Some delay due to the internal reorganisation and administrative procedures for personnel recruitment. Transfer of the algorithm and related documentation took more time than expected due to authorization procedures at the oGP site. The order of actions under Activity 2 changed after technical evaluations	Monitoring of the recruitment procedures and collaboration with the administrative offices to speed up the process. Reorder of the activities while waiting the algorithm.	Personnel was successfully recruited. The new order of the activities increased the efficiency of the plan.
	Availability of IT infrastructure (Y/N)	Yes	N.A. (Expected during PDSA 2)			
	% of data processed	100%	N.A. (Expected during PDSA 2)			
**LCF 1 – Activity 3:** Define care programs and services for each of the strata	Short report on care programs and services (Y/N)	Yes	N.A. (Expected during PDSA 2)	Not yet started		

1.1.2.1.4 Act

Cycle number (1 or 2)	1		
**Activity**	**Maintain**	**Adapt**	**Abandon**
**LCF 1 – Activity 1:** Identify criteria for local stratification model	X		
**LCF 1 – Activity 2:** Implement data extraction and processing		X - The order of the actions needs to be changed due to technical	

mechanisms			
**LCF 1 – Activity 3:** Define care programs and services for each of the strata		X - Timing of the actions needs to be extended. Alignment with National/regional guidelines currently in progress is needed.	

QUESTIONS	ANSWERS
**Any new proposed action for the future?**	Pilot testing of GMA implementation

1.1.2.2 2nd PDSA Cycle

1.1.2.2.1 Plan

**LCF1**	**Implement a risk stratification tool based on adjusted morbidity group (GMA)**							
**Activities (from the LAP)**	**Actions**	**Actors**	**Timeline**	**KPIs measure (data collection)**				
				**KPI**	**Who**	**When**	**How**	**Target**
Activity 1: Implement data extraction and processing mechanisms	Automating the data extraction and validation process	-Data analysts<br>-IT experts<br>-Technicians Healthcare professionals	01/04/22 to 31/08/22	1.Database with required health data (Y/N)<br>2.Short report on quality check (Y/N)<br>3.Procedure for data extraction (Y/N)<br>4. Short report on pilot test results (Y/N)<br>5.Integration of the tool in the regional IT infrastructure (Y/N)	Project manager<br>Data Analyst	31/08/2022	Utilising information technology systems<br>Monitoring during the weekly/monthly follow-up meetings	1.Yes<br>2.Yes<br>3.Yes<br>4.Yes<br>5.Yes
	Standardize variables format based on algorithm for stratification					30/09/2022		
	Pilot test of algorithm					30/11/2022		
	Implement algorithm tool					31/01/2023		
Activity 2: Define care programs and services for each of the strata	Elaborate population management strategies for GMA strata, starting with health promotion, to case management strategy, focusing on chronic diseases	-Healthcare professionals<br>-Marche/ARS staff	01/09/22 to 30/11/22	Guidelines on population management strategies (Y/N)	Project manager	30/11/22	Relying on specific evaluation and guidance from healthcare professionals Monitoring during the monthly follow-up meetings	Yes

	management							

LCF2	Build a map/dashboard of citizens’ health/risk and available services							
**Activities (from the LAP)**	**Actions**	**Actors**	**Timeline**	**KPIs measure (data collection)**				
				**KPI**	**Who**	**When**	**How**	**Target**
Activity 1: Definition of indicators on stratification and/or chronic diseases	Mapping of relevant indicators at National/Regional levels	-Healthcare professionals<br>-Data analysts<br>-IT experts<br>-Marche/ARS staff	01/09/22 to 30/09/22	List of indicators (Y/N)	Project manager	30/09/22<br>31/10/22	Monitoring during the weekly/monthly follow-up meetings	Yes
	Development of new/revision of existing indicators		01/10/22 to 31/10/22					
Activity 2: Mapping of available services for chronic diseases in Marche Region	Collection of databases/registries of health and social care facilities/services (e.g. community services, hospitals, residential and semi-residential facilities), including info on sources, timing of updates, classification and users	Marche/ARS staff	01/09/22 to 30/11/22	List of facilities/services (Y/N)	Project manager	30/11/22	Monitoring during the weekly/monthly follow-up meetings	Yes
Activity 3: Set up of the dashboard for data visualization	Revision of available platforms and definition of technical requirements	-Marche/ARS staff<br>-Healthcare professionals	01/07/22 to 30/09/22	Availability of the dashboard (Y/N)	Project manager	31/12/22	Monitoring during the weekly/monthly follow-up meetings	Yes
	Implementation of the		01/10/22 to					

	dashboard	-Data analysts<br>-IT experts	31/12/22					
Activity 4: Identification of policies and interventions at regional level to support implementation and sustainability of the LGP	Monitoring and analysis of relevant National/Regional guidelines, laws, plans	-Marche/ARS staff<br>-Healthcare professionals<br>-Data analysts<br>-Regional policy representatives	01/04/22 to 30/09/23	Sustainability plan (Y/N)	Project manager	31/12/22	Monitoring during the monthly follow-up meetings	Yes
	Definition of a sustainability plan for the integration of the tool in the decision-making process		01/09/22 to 31/12/22					

1.1.2.2.2 Do

Cycle number (1 or 2)	2	
**Activity**	**KPI**	**Actual value**
LCF 1 - Activity 1: Implement data extraction and processing mechanisms	Database with required health data (Y/N)	Yes
	Short report on quality check (Y/N)	Yes
	Procedure for data extraction (Y/N)	Yes
	Short report on pilot test results (Y/N)	Yes
	Integration of the tool in the regional IT infrastructure (Y/N)	Yes (partially)
LCF 1 - Activity 2: Define care programs and services for each of the strata	Guidelines on population management strategies (Y/N)	Yes
LCF 2 - Activity 1: Definition of indicators on stratification and/or chronic diseases	List of indicators (Y/N)	Yes
LCF 2 - Activity 2: Mapping of available services for chronic diseases in Marche Region	List of facilities/services (Y/N)	Yes
LCF 2 - Activity 3: Set up of the dashboard for data visualization	Availability of the dashboard (Y/N)	No (Ongoing, preliminary structure of the dashboard available)
LCF 2 - Activity 4: Identification of policies and interventions at regional level to support implementation and sustainability of the LGP	Sustainability plan (Y/N)	Yes

QUESTIONS	ANSWERS
**What was actually implemented? Any deviation from the planned actions**	Main achievements of PDSA2 are:<br><br>- complete database of regional population, through the linkage of administrative health databases (Hospital discharges, Emergency & Urgency, Home care, Exemptions, population registry)

	- quality evaluation of health data with identification of improvement actions (through quality reports and experts' opinion)<br>- report on population stratification, gained through GMA's first application.<br>- structure of a dashboard for indicators and data visualization<br>- guidelines of population management's strategies, through new National/Regional regulations<br><br>Some deviations occurred: there were some delays due to the complexity of the data preparation phase. Administrative health care databases' analysis and linkage took more time than expected due to their complexity and to the need of conducting data quality evaluation with health databases' experts. For this reason, the integration with the Regional IT system was delayed but partially achieved (completion expected in one month). Ongoing reorganisation of the regional healthcare system had an impact on some tasks due to overburdened personnel and for the ongoing changes in the IT systems.<br><br>The setting up of dashboard is complex, in terms of statistical computing and clinical relevance. Previous plans on reusing internal tools were abandoned (for the time being) because of changes in the organisation and time restrictions. Dashboard and indicators are being defined, also with the support of the oGP but the implementation is still ongoing, and it will continue during the post-implementation phase.
**Problems? Unexpected findings? Please describe**	Some challenges occurred due to the data complexity. Indeed, the management of the administrative health databases is challenging due to privacy restrictions, authorizations, IT issues. However, these issues are constantly monitored and partially solved. Main barrier to the full implementation of the tool is the GDPR application in Italy, still under debate.<br><br>As regards unexpected findings, we can acknowledge the great interest of healthcare managers in the project and their fruitful collaboration during the quality check and indicators/dashboard development phases. Indeed, quality data evaluation showed the need to conduct further analysis and insights of healthcare databases. Moreover, the fruitful discussions enhanced the identification of relevant aspects for healthcare services programming which will be considered in the next implementation period.<br><br>The data preparation phase was very challenging but, in the end, the results achieved confirmed the great potential of the tool. Moreover, we succeed to include the preliminary results of the algorithm in a Regional Deliberation on the adoption of the new National standards of community healthcare services, as a part of the regional implementation of the Recovery and Resilience Plan.

IMPLEMENTATION PROGRESS OF THE LOCAL GOOD PRACTICE			
**0-25%**	**25-50%**	**50-75%**	**75-100%**
			**x**

1.1.2.2.3 Study

**Cycle number**		**2**				
**Activity**	**KPI**	**Target value**	**Actual value**	**Reasons for the deviations**	**Mitigation actions implemented**	**Impact of mitigation actions**
LCF 1 - Activity 1: Implement data extraction and processing mechanisms	Database with required health data (Y/N)	Yes	Yes			
	Short report on quality check (Y/N)	Yes	Yes			
	Procedure for data extraction (Y/N)	Yes	Yes			
	Short report on pilot test results (Y/N)	Yes	Yes			
	Integration of the tool in the regional IT infrastructure (Y/N)	Yes	Yes (partially)	Delay due to longer time needed to complete previous activities and overburdened personnel	Reorganization of the activities	Gain time to set up the server
LCF 1 - Activity 2: Define care	Guidelines on population management strategies	Yes	Yes			

programs and services for each of the strata	(Y/N)					
LCF 2 - Activity 1: Definition of indicators on stratification and/or chronic diseases	List of indicators (Y/N)	Yes	Yes			
LCF 2 - Activity 2: Mapping of available services for chronic diseases in Marche Region	List of facilities/services (Y/N)	Yes	Yes			
LCF 2 - Activity 3: Set up of the dashboard for data visualization	Availability of the dashboard (Y/N)	Yes	No (Ongoing, preliminary structure of the dashboard available)	Delays in the data preparation phase; need to revise previously planned plans	New option under development	Enhancing implementation of the activity
LCF 2 - Activity 4: Identification of policies and interventions at regional level to support implementation and	Sustainability plan (Y/N)	Yes	Yes			

sustainability of the LGP						

1.1.2.2.4 Act

**Cycle number (1 or 2)**	**2**		
**Activity**	**Maintain**	**Adapt**	**Abandon**
LCF 1 - Activity 1: Implement data extraction and processing mechanisms		**X -** Additional activities are foreseen in the following months to: revise and simplify data preparation procedure; complete integration into IT system	
LCF 1 - Activity 2: Define care programs and services for each of the strata	**X -** Activity completed. Need to be monitored according to reorganization of the healthcare system and new regulations on healthcare services.		
LCF 2 - Activity 1: Definition of indicators on stratification and/or chronic diseases	**X -** Activity completed. Need to be monitored to address new needs of healthcare managers and policymakers according to National requirements and reorganization of the Regional healthcare system.		
LCF 2 - Activity 2: Mapping of available services for chronic diseases in Marche Region	**X -** Activity completed. Need to be monitored according to reorganization of the healthcare system and new regulations on healthcare services.		
LCF 2 - Activity 3: Set up of the dashboard for data visualization		**X -** Timing of the actions needs to be extended, to analyse in detail different options of data visualization and find the most suitable solution for our context. It is foreseen the prosecution of the activity in the following	

		months.	
LCF 2 - Activity 4: Identification of policies and interventions at regional level to support implementation and sustainability of the LGP	**X** **-** Activity continuously progressing.		

QUESTIONS	ANSWERS
**Any new proposed action for the future?**	TECHNICAL/OPERATIVE ACTIONS:<br>• *Improvements in technical and graphic part of the dashboard*, also through development of maps to visualize healthcare services that will be activated based on recent social and health planning regulations (at national and regional level).<br>• *Revision of Indicators in alignment with the needs of experts in the various healthcare sectors,* e.g. through regular updating, and evaluation of their presentation modality at a higher level of detail (if possible, according to GDPR regulation).<br>• *Addition of further healthcare databases for AMG and indicators' computing (e.g.: hospice database)*, to make the data more accurate and to add information potentially relevant.<br>• *Make it a useful tool for regional and clinical managers* for close monitoring of population's health status and resources consumption, with possible comparison and benchmarking activities.<br>• Implementing of the dashboard in order to comply with the requirements of recent national and regional regulations regarding the *stratification mandate.*<br><br>SUSTAINABILITY ACTIONS:<br>• Enhancing and maximising AMG and dashboard's potential through *definition of a roadmap* to enhance its use as a tool for regional and clinical managers, e.g., for programming and implementing healthcare policies and services, process of budget and resources application, supporting investment in prevention and continuity of care (such as territorial operative centres, community hospitals and houses, home care, family nursing, palliative care, and telemedicine)<br>• Defining further analysis and actions to improve the quality and completeness of healthcare data.<br>Disseminating data culture in institutions, raising awareness of the health care professionals on proper reporting of information

1.2 Estonia (EE)

1.2.1 Local Action Plan

1.2.1.1 Local Good Practice

<table>
<tr><th>Local Good Practice</th><td colspan="2">A funding model for person-centred and integrated services</td></tr>
<tr><th colspan="2">Target population</th><th>Setting(s)</th></tr>
<tr><td colspan="2">~50,000 habitants</td><td>Viljandi county</td></tr>
<tr><th colspan="3">Main aim</th></tr>
<tr><td colspan="3">Improve the results of the health and quality of life of the population and increase the efficiency of the healthcare system through better planning and use of resources.</td></tr>
<tr><th>Outcomes</th><th>Local Core Features and their Components</th><th>Inputs</th></tr>
<tr><td>• Cooperation between Viljandi Hos pital and other service providers is carried out.
• IT tools supporting integrated care funding modelling and risk stratification.
• Funding model has been proposed in integrated care provision in Viljandi county.
• Assessment feasibility of nationwide implementation of oGP-s.</td><td>Develop a funding model for person-centred and integrated services:

• Risk stratification model
• Case finding
• Value-based contracting and payment framework
• Analytical model to execute the contract.</td><td>• Assessment of transferability of OptiMedis framew ork.
• Assessment of transferability of risk stratification and case finding tools.
• Identification of steps for adoption of the Catalan population-based risk stratification tool into the ecosystem of the NA.</td></tr>
<tr><th colspan="3">General description</th></tr>
<tr><td colspan="3">Generating predictive model is needed to strengthen population health management and provide better-tailored services for risk groups. Contracting and funding models developed are lined with person-centred and integrated services.</td></tr>
<tr><th colspan="3">Local Core Feature 1</th></tr>
<tr><td colspan="3">Develop a funding model for person-centred and integrated services:
• Risk stratification model.
• Case finding.
• Value-based contracting and payment framework.
• Analytical model to execute the contract.</td></tr>
</table>

1.2.1.2 Local Action Plan

Local Good Practice	A funding model for person-centred and integrated services
**Target population**	**Setting**
~50,000 habitants	Viljandi county
**Main aim**	
Improve the results of the health and quality of life of the population and increase the efficiency of the healthcare system through better planning and use of resources.	
**General description**	
Generating predictive model is needed to strengthen population health management and provide better-tailored services for risk groups. Contracting and funding models developed are lined with person-centred and integrated services.	
**Related oGPs and CFs**	Mix'n'Match OptiMedis & Catalan OGP<br><br>CF1.1 Assessment of transferability, and identification of steps for adoption, according to intellectual property rules, of the Catalan population-based risk stratification tool (AMG) into the ecosystem of the next adopter.<br>CF1.2 Health data management strategies<br>CF1.3 Development of enhanced risk prediction modelling for health policy purposes and/or clinical risk prediction
**Local Core Feature 1**	**Develop a funding model for person-centred and integrated services.**
**SMART objective**	
We will design a contracting and payment framework approach based on OptiMedis that includes Catalonian AMG risk stratification model.	

Activities	Actors	Resources	Setting(s)	Timeline	KPIs
Create a core group to define the local contracting and payment framework model	-GPs, nurses<br>-Hospital doctors and nurses<br>-Healthcare planning experts	Professionals from different settings	-GP practices<br>-Viljandi Hospital<br>-Estonian Ministry of Social Affairs	01.01.2022,<br>(5 months)	Number and profile of professionals engaged in the definition of the contracting and payment framework approach.
Establish criteria for contracting and payment framework	-Healthcare professionals<br>-OptiMedis experts<br>-AMG experts	Professionals from different settings	-Viljandi Hospital<br>-Estonian Ministry of Social Affairs	02.02.2022<br>(4 months)	List of criteria used for contracting and payment framework (Y/N)

			-EHIF		
Set up the data extraction and processing mechanisms	-IT experts<br>-Data scientists<br>-OptiMedis experts<br>-AMG experts	-OptiMedis experts<br>-AMG experts<br>-IT infrastructure<br>-Subcontractor for technical development	Viljandi Hospital	01.01.2022<br>(5 months)	-Database creation (Y/N)<br>-Technical design (%)<br>-Functional design (%)
Implement case finding and risk stratification	-IT experts<br>-Data scientists<br>-OptiMedis experts<br>-AMG experts	-IT infrastructure<br>-Subcontractor for technical development	Viljandi Hospital	01.04.2022<br>(5 months)	Case finding and risk stratification tool<br>is implemneted (Y/N)
Design contracting and payment framework	-GPs<br>-Hospital management.<br>-OptiMedis experts<br>-AMG experts	Professionals from different settings	-Viljandi Hospital<br>-Estonian Ministry of Social Affairs<br>-EHIF	01.04.2022<br>(5 months)	Contracting and payment framework agreed (Y/N)
Assess case finding and risk stratification based contracting and payment framework against established cri	-Experts<br>-Healthcare professionals<br>-Healthcare planning	Professionals from different settings	-Viljandi Hospital<br>-GP practices<br>-Estonian	01.09.2022<br>(3 months)	Conformance report (Y/N)

teria	experts		Ministry of Social Affairs<br><br>-EHIF		

1.2.2 Plan-Do-Study-Act Cycles

1.2.2.1 1st PDSA Cycle

1.2.2.1.1 Plan

**LCF1**	**Develop a funding model for person-centred and integrated services.**							
**Activities (from the LAP)**	**Actions**	**Actors**	**Timeline**	**KPIs measure (data collection)**				
				**KPI**	**Who**	**When**	**How**	**Target**
Activity 1: Create a core group to define the local contracting and payment framework model	Reaching core stakeholders agreement	-GPs, nurses.<br><br>-Hospital doctors and nurses.<br><br>-Healthcare planning experts.	01.01.22 - 31.01.22	Number and profile of professionals engaged in the definition of the contracting and payment framework approach	VH project manager	On stakeholder's meetings	Registration forms	5
	Expanding and agreeing with key stakeholders	-GPs, nurses.<br><br>-Hospital doctors and nurses.<br><br>-Healthcare planning experts	01.02.22 - 28.02.22					
	Agreeing on all stakeholder's letter of intent of Viljandi county	-GPs, nurses.<br><br>-Hospital doctors and nurses.	01.03.22 - 31.03.22					

		-Healthcare planning experts						
Activity 2: Establish criteria for contracting and payment framework	Introduction and creating possible scenarios	-IT Experts<br><br>-Healthcare professionals	02.02.22<br>-<br>28.02.22	List of criteria used for contracting and payment framework (Y/N)	VH project manager	On workshop	Agreement	Yes
	Agreeing roadmap	-IT Experts<br><br>-Healthcare professionals	01.03.22<br>-<br>31.03.22					
	Defining alternatives	-IT Experts<br><br>-Healthcare professionals	01.04.22<br>-<br>30.04.22					
	Concluding agreement	-IT Experts<br><br>-Healthcare professionals	01.05.22<br>-<br>31.05.22					
Activity 3: Set up the data extraction and processing mechanisms.	Improving incrementally data extract and loading (ETL) according to model requirements - Phase 1	-IT experts<br><br>-Data scientists	01.01.22<br>-<br>31.01.22	1.Database creation (Y/N)<br><br>2.Technical design (%)<br><br>3.Functional design (%)	VH project manager	After Phase 5 data extract and loading	Phase 5 report on data extract and loading	1.Yes<br><br>2.100%<br><br>3.100%
	Improving incrementally data extract and loading (ETL) - Phase 2	-IT experts<br><br>-Data scientists	01.02.22<br>-<br>28.02.22					
	Improving incrementally data extract and loading (ETL) -	-IT experts	01.03.22<br>-					

	Phase 3	-Data scientists	31.03.22					
	Improving incrementally data extract and loading (ETL) - Phase 4	-IT experts<br><br>-Data scientists	01.04.22<br>-<br>30.04.22					
	Improving incrementally data extract and loading (ETL) - phase 5	-IT experts<br><br>-Data scientists	01.05.22<br>-<br>31.05.22					
Activity 4: Implement case finding and risk stratification.	Gathering and systematising LAP specific information and planning further action based upon agreed roadmap	-IT experts<br><br>-Data scientists	01.4.22-<br>30.4.22	Case finding and risk stratification tool<br>is implemented (Y/N)	VH project manager	After publishing local case finding and risk stratificatio n framework	Published local case finding and risk stratificatio n framework	Yes
	Drafting local case finding and risk stratification framework	-IT experts<br><br>-Data scientists	01.05.22<br>-31.5.22					
	Drafting local case finding and risk stratification framework	-IT experts<br><br>-Data scientists	01.06.22<br>-<br>30.06.22					
	Agreeing on local case finding and risk stratification framework	-IT experts<br><br>-Data scientists	01.08.22<br>-<br>31.08.22					
Activity 5:<br>Design contracting and payment framework.	Gathering and systematising LAP specific information and planning further action based	-IT Experts<br><br>-Healthcare	01.4.22-<br>30.4.22	Contracting and payment framework proposed (Y/N)	VH project manager	After publishing local	Published local contracting	Yes

	upon agreed roadmap	professionals<br><br>-Healthcare planning experts				contracting and payment framework	and payment framework	
	Drafting local contracting and payment framework	-IT Experts<br><br>-Healthcare professionals<br><br>-Healthcare planning experts	01.05.22 -31.5.22					
	Improving local contracting and payment framework	-IT Experts<br><br>-Healthcare professionals<br><br>-Healthcare planning experts	01.06.22 - 30.06.22					
	Agreeing on local contracting and payment framework	-IT Experts<br><br>-Healthcare professionals<br><br>-Healthcare planning experts	01.08.22 - 31.08.22					
Activity 6:<br>Assess proposed case	Introducing preliminary report and gathering feedback	-IT Experts	01.09.22 -	Conformance report (Y/N)	VH project	After publishing	Published stakeholder	Yes

finding and risk stratification based contr acting and payment framework against estab lished criteria.		-Healthcare professionals<br><br>-Healthcare planning experts	30.09.22		manager	stakeholder s contracting and payment framework model agreement	s contracting and payment framework model agreement	
	Introducing improved report and gathering feedback	-IT Experts<br><br>-Healthcare professionals<br><br>-Healthcare planning experts	01.10.22 - 31.10.22					
	Introducing agreement draft and gathering feedback and finalizing report and agreement	-IT Experts<br><br>-Healthcare professionals<br><br>-Healthcare planning experts	01.11.22 - 30.11.22					

1.2.2.1.2 Do

Cycle number (1 or 2)	1	
**Activity**	**KPI**	**Actual value**
**LCF 1 - Activity 1:** Create a core group to define the local contracting and payment framework model	Number and profile of professionals engaged in the definition of the contracting and payment framework approach	5
**LCF 1 - Activity 2:** Establish criteria for contracting and payment framework	List of criteria used for contracting and payment framework (Y/N)	Yes
**LCF 1 - Activity 3:** Set up the data extraction and processing mechanisms.	Database creation (Y/N)	Yes
	Technical design (%)	100%
	Functional design (%)	100%
**LCF 1 - Activity 4:** Implement case finding and risk stratification.	Case finding and risk stratification tool is implemented (Y/N)	To be completed in PDSA 2
**LCF 1 - Activity 5**: Design contracting and payment framework.	Contracting and payment framework proposed (Y/N)	To be completed in PDSA 2
**LCF 1 – Activity 6:** Assess proposed case finding and risk stratification based contracting and payment framework against established criteria.	Conformance report (Y/N)	To be completed in PDSA 2

QUESTIONS	ANSWERS
**What was actually implemented? Any deviation from the planned actions**	Health care professionals from primary care & hospital care are engaged (GPs, specialist doctors, RN both family nurses and hospital and home care nurses, social workers, data manger), planned no (5) was exceeded for synergy of combined expertise. There is a list of criteria of the contracting and payment framework approach created. Database is created and design requirements for technical and functionality full-filled.
**Problems? Unexpected findings? Please describe**	Due to current situation in health care has slowed down the planned activities, meetings have been held, plans expanded and agreed with key stakeholders discussed.

IMPLEMENTATION PROGRESS OF THE LOCAL GOOD PRACTICE			
**0-25%**	**25-50%**	**50-75%**	**75-100%**
	**X**		

1.2.2.1.3 Study

Cycle number		1				
**Activity**	**KPI**	**Target value**	**Actual value**	**Reasons for the deviations**	**Mitigation actions implemented**	**Impact of mitigation actions**

**LCF 1 - Activity 1:** Create a core group to define the local contracting and payment framework model	Number and profile of professionals engaged in the definition of the contracting and payment framework approach	5	5			
**LCF 1 - Activity 2:** Establish criteria for contracting and payment framework	List of criteria used for contracting and payment framework (Y/N)	Yes	Yes			
**LCF 1 - Activity 3:** Set up the data extraction and processing mechanisms.	Database creation (Y/N)	Yes	Yes			
	Technical design (%)	100%	100%			
	Functional design (%)	100%	100%			
**LCF 1 - Activity 4:** Implement case finding and risk stratification.	Case finding and risk stratification tool is implemented (Y/N)	Yes	To be completed in PDSA 2			
**LCF 1 - Activity 5:** Design contracting and payment framework.	Contracting and payment framework proposed (Y/N)	Yes	To be completed in PDSA 2			
**LCF 1 – Activity 6:** Assess proposed case finding and risk stratification based contracting and payment framework against established criteria.	Conformance report (Y/N)	Yes	To be completed in PDSA 2			

1.2.2.1.4 Act

**Cycle number (1 or 2)**	**1**		
**Activity**	**Maintain**	**Adapt**	**Abandon**
**LCF 1 - Activity 1:** Create a core group to define the local contracting and payment framework model	**X**		
**LCF 1 - Activity 2:** Establish criteria	**X**		

for contracting and payment framework			
**LCF 1 - Activity 3:** Set up the data extraction and processing mechanisms.	**X**		
**LCF 1 - Activity 4:** Implement case finding and risk stratification.	**X**		
**LCF 1 - Activity 5:** Design contracting and payment framework.	**X**		
**LCF 1 – Activity 6:** Assess proposed case finding and risk stratification based contracting and payment framework against established criteria.	**X**		

QUESTIONS	ANSWERS
**Any new proposed action for the future?**	oGP expertise is present and available; national stakeholders are interested; discussions are in place to define synergies between partners and opportunities

1.2.2.2 2nd PDSA Cycle

1.2.2.2.1 Plan

**LCF1**	**Develop a funding model for person-centred and integrated services.**							
**Activities (from the LAP)**	**Actions**	**Actors**	**Timeline**	**KPIs measure (data collection)**				
				**KPI**	**Who**	**When**	**How**	**Target**
Activity 1: Create a core group to define the local contracting and payment framework model	Reaching core stakeholders agreement	-GPs, nurses.<br><br>-Hospital doctors and nurses.<br><br>-Healthcare planning experts.	01.01.22 - 31.01.22	Number and profile of professionals engage d in the definition of the contracting and payment framework approach	VH project manager	On stakeholder's meetings	Registration forms	5
	Expanding and agreeing with key stakeholders	-GPs, nurses.<br><br>-Hospital doctors and nurses.<br><br>-Healthcare planning experts	01.02.22 - 28.02.22					
	Agreeing on all stakeholder's letter of intent of Viljandi county	-GPs, nurses.<br><br>-Hospital doctors and nurses.<br><br>-Healthcare	01.03.22 - 31.03.22					

		planning experts						
Activity 2: Establish criteria for contracting and payment framework	Introduction and creating possible scenarios	-IT Experts<br><br>-Healthcare professionals	02.02.22<br>-<br>28.02.22	List of criteria used for contracting and payment framework (Y/N)	VH project manager	On workshop	Agreement	Yes
	Agreeing roadmap	-IT Experts<br><br>-Healthcare professionals	01.03.22<br>-<br>31.03.22					
	Defining alternatives	-IT Experts<br><br>-Healthcare professionals	01.04.22<br>-<br>30.04.22					
	Concluding agreement	-IT Experts<br><br>-Healthcare professionals	01.05.22<br>-<br>31.05.22					
Activity 3: Set up the data extraction and processing mechanisms.	Improving incrementally data extract and loading (ETL) according to model requirements - Phase 1	-IT experts<br><br>-Data scientists	01.01.22<br>-<br>31.01.22	1.Database creation (Y/N)<br><br>2.Technical design (%)<br><br>3.Functional design (%)	VH project manager	After Phase 5 data extract and loading	Phase 5 report on data extract and loading	1.Yes<br><br>2.100%<br><br>3.100%
	Improving incrementally data extract and loading (ETL) - Phase 2	-IT experts<br><br>-Data scientists	01.02.22<br>-<br>28.02.22					
	Improving incrementally data extract and loading (ETL) - Phase 3	-IT experts<br><br>-Data	01.03.22<br>-<br>31.03.22					

		scientists						
	Improving incrementally data extract and loading (ETL) - Phase 4	-IT experts<br><br>-Data scientists	01.04.22<br>-<br>30.04.22					
	Improving incrementally data extract and loading (ETL) - phase 5	-IT experts<br><br>-Data scientists	01.05.22<br>-<br>31.05.22					
Activity 4: Implement case finding and risk stratification.	Gathering and systematising LAP specific information and planning further action based upon agreed roadmap	-IT experts<br><br>-Data scientists	01.4.22-<br>30.4.22	Case finding and risk stratification tool is implemented (Y/N)	VH project manager	After publishing local case finding and risk stratificatio n framework	Published local case finding and risk stratificatio n framework	Yes
	Drafting local case finding and risk stratification framework	-IT experts<br><br>-Data scientists	01.05.22<br>-31.5.22					
	Drafting local case finding and risk stratification framework	-IT experts<br><br>-Data scientists	01.06.22<br>-<br>30.06.22					
	Agreeing on local case finding and risk stratification framework	-IT experts<br><br>-Data scientists	01.08.22<br>-<br>31.08.22					
Activity 5:<br>Design contracting and payment framework.	Gathering and systematising LAP specific information and planning further action based upon agreed roadmap	-IT Experts<br><br>-Healthcare professionals	01.4.22-<br>30.4.22	Contracting and payment framework proposed (Y/N)	VH project manager	After publishing local contracting	Published local contracting and	Yes

		-Healthcare planning experts				and payment framework	payment framework	
	Drafting local contracting and payment framework	-IT Experts<br><br>-Healthcare professionals<br><br>-Healthcare planning experts	01.05.22 -31.5.22					
	Improving local contracting and payment framework	-IT Experts<br><br>-Healthcare professionals<br><br>-Healthcare planning experts	01.06.22 - 30.06.22					
	Improving local contracting and payment framework based on CPTS contracting and payment framework	-IT Experts<br><br>-Healthcare professionals<br><br>-Healthcare planning experts	01.08.22 - 31.08.22					
Activity 6:<br>Assess proposed case finding and risk	Introducing preliminary report and gathering feedback	-IT Experts<br><br>-Healthcare	01.09.22 - 30.09.22	Conformance report (Y/N)	VH project manager	After publishing stakeholder	Published stakeholder s	Yes

stratification based contracting and payment framework against established criteria.		professionals<br><br>-Healthcare planning experts				s contracting and payment framework model agreement	contracting and payment framework model agreement	
	Introducing improved report and gathering feedback	-IT Experts<br><br>-Healthcare professionals<br><br>-Healthcare planning experts	01.10.22<br>-<br>31.10.22					
	Introducing agreement draft and gathering feedback.<br>Finalizing report and agreement	-IT Experts<br><br>-Healthcare professionals<br><br>-Healthcare planning experts	01.11.22<br>-<br>30.11.22					

1.2.2.2.2 Do

Cycle number (1 or 2)	2	
**Activity**	**KPI**	**Actual value**
**LCF 1 - Activity 1**: Create a core group to define the local contracting and payment framework model	Number and profile of professionals engaged in the definition of the contracting and payment framework approach	5
**LCF 1 - Activity 2:** Establish criteria for contracting and payment framework	List of criteria used for contracting and payment framework (Y/N)	Yes
**LCF 1 - Activity 3:** Set up the data extraction and processing mechanisms.	Database creation (Y/N)	Yes
	Technical design (%)	100%
	Functional design (%)	100%
**LCF 1 - Activity 4:** Implement case finding and risk stratification.	Case finding and risk stratification tool is implemented (Y/N)	Yes
**LCF 1 - Activity 5:** Design contracting and payment framework.	Contracting and payment framework proposed (Y/N)	Yes
**LCF 1 – Activity 6:** Assess proposed case finding and risk stratification based contracting and payment framework against established criteria.	Conformance report (Y/N)	Yes

QUESTIONS	ANSWERS
**What was actually implemented? Any deviation from the planned actions**	Case finding and risk stratification tool is used locally, sustainability actions are planned to implement the tool at national level (national project "PAIK2022-2025" initiated). Contracting and payment framework agreed among the current project team, discussions with stakeholders done and framework implementation will follow over some time of the period.
**Problems? Unexpected findings? Please describe**	Regarding the contracting and payment framework model implementation on municipality level - the local level stakeholders & collaborative partner active involvement is slightly slowed down due to the raised workload related to their usual tasks, and the change of contact person; stakeholders contracting and payment framework model agreement reporting delayed.

IMPLEMENTATION PROGRESS OF THE LOCAL GOOD PRACTICE			
**0-25%**	**25-50%**	**50-75%**	**75-100%**

			**X**

1.2.2.2.3 Study

**Cycle number**		**2**				
**Activity**	**KPI**	**Target value**	**Actual value**	**Reasons for the deviations**	**Mitigation actions implemented**	**Impact of mitigation actions**
**LCF 1 - Activity 1:** Create a core group to define the local contracting and payment framework model	Number and profile of professionals engaged in the definition of the contracting and payment framework approach	5	5			
**LCF 1 - Activity 2:** Establish criteria for contracting and payment framework	List of criteria used for contracting and payment framework (Y/N)	Yes	Yes			
**LCF 1 - Activity 3:** Set up the data extraction and processing mechanisms.	Database creation (Y/N)	Yes	Yes			
	Technical design (%)	100%	100%			
	Functional design (%)	100%	100%			
**LCF 1 - Activity 4:** Implement case finding and risk stratification.	Case finding and risk stratification tool is implemented (Y/N)	Yes	Yes			
**LCF 1 - Activity 5:** Design contracting and payment framework.	Contracting and payment framework proposed (Y/N)	Yes	Yes			
**LCF 1 – Activity 6:** Assess proposed case finding and	Conformance report (Y/N)	Yes	Yes			

risk stratification bas ed contracting and payment framework again st established cri teria.						

1.2.2.2.4 Act

Cycle number (1 or 2)	2		
**Activity**	**Maintain**	**Adapt**	**Abandon**
**LCF 1 - Activity 1:** Create a core group to define the local contracting and payment framework model	**X**		
**LCF 1 - Activity 2:** Establish criteria for contracting and payment framework	**X**		
**LCF 1 - Activity 3:** Set up the data extraction and processing mechanisms.	**X**		
**LCF 1 - Activity 4:** Implement case finding and risk stratification.	**X**		
**LCF 1 - Activity 5:** Design contracting and payment framework.	**X**		
**LCF 1 – Activity 6:** Assess proposed case finding and risk stratification based contracting and payment framework against established criteria.	**X**		

QUESTIONS	ANSWERS
**Any new proposed action for the future?**	No completely new activities proposed but some oGP support while ensuring the sustainability (both on regional and national level) of already planned and tested activities might be beneficial.

APPENDIX 2: JADECARE THEMATIC WORKSHOP, LECTURE ON HEALTH RISK ASSESSMENT, DAVID MONTERDE - VILJANDI 14TH JUNE 2022

Link to access the recording of the lecture on health risk assessment given by David Monterde in Viljandi (14th June 2022) in the context of the health risk assessment workshop as part of the activity of the EU project JADECARE:

https://drive.google.com/drive/folders/1Dwx2C2Ek2Z3mSz-2MRCJepWmzDrEggdh?usp=sharing
